# Identification and Classification of Rhizobia by Matrix-Assisted Laser Desorption/Ionization Time-Of-Flight Mass Spectrometry

**DOI:** 10.4172/jpb.1000357

**Published:** 2015-05-31

**Authors:** Rui Zong Jia, Rong Juan Zhang, Qing Wei, Wen Feng Chen, Il Kyu Cho, Wen Xin Chen, Qing X Li

**Affiliations:** 1 Department of Molecular Biosciences and Bioengineering, University of Hawaii at Manoa, Honolulu, HI 96822, USA; 2 State Key Laboratory of Agro biotechnology, College of Biological Sciences, China Agricultural University, Beijing, 100193, China; 3 State Key Biotechnology Laboratory for Tropical Crops, Institute of Tropical Bioscience and Biotechnology, Chinese Academy of Tropical Agriculture Sciences, Haikou, Hainan, 571101, China; 4 Dongying Municipal Bureau of Agriculture, Dongying, Shandong, 257091, China

**Keywords:** Bacterial identification, Bacterial classification, MALDI TOF MS, *Rhizobium*

## Abstract

Mass spectrometry (MS) has been widely used for specific, sensitive and rapid analysis of proteins and has shown a high potential for bacterial identification and characterization. Type strains of four species of rhizobia and *Escherichia coli* DH5α were employed as reference bacteria to optimize various parameters for identification and classification of species of rhizobia by matrix-assisted laser desorption/ionization time-of-flight MS (MALDI TOF MS). The parameters optimized included culture medium states (liquid or solid), bacterial growth phases, colony storage temperature and duration, and protein data processing to enhance the bacterial identification resolution, accuracy and reliability. The medium state had little effects on the mass spectra of protein profiles. A suitable sampling time was between the exponential phase and the stationary phase. Consistent protein mass spectral profiles were observed for *E. coli* colonies pre-grown for 14 days and rhizobia for 21 days at 4°C or 21°C. A dendrogram of 75 rhizobial strains of 4 genera was constructed based on MALDI TOF mass spectra and the topological patterns agreed well with those in the 16S rDNA phylogenetic tree. The potential of developing a mass spectral database for all rhizobia species was assessed with blind samples. The entire process from sample preparation to accurate identification and classification of species required approximately one hour.

## Introduction

Species of rhizobia are unique bacteria due to their symbiotic relationship with legumes. Rhizobia colonize host legume plants and convert atmospheric nitrogen (N_2_) into ammonia (NH_3_) to provide nitrogen nutrients for both rhizobia and the host plant [1,2]. This nitrogen-fixing ability makes rhizobia most valuable resource in natural and agricultural ecosystems, which plays a key role in the nitrogen cycle. Biological nitrogen fixation (BNF) accounted for 65% of global nitrogen resources (in term of mineral, not N_2_ gas), while chemical syntheses made about 30% [2]. Three quarters of BNF are generated through symbiosis between rhizobia and legume plants.

The host specificity for rhizobia involves a finely-tuned signal exchange between the host and its rhizobial partner [3]. Although the symbiotic genes are similar, there are wide variations in nodulation and host effects. Species of rhizobia are host-specific. Different species of rhizobia have difference legume hosts and different rhizobial isolates in the same species have different BNF efficiencies. In other words, rhizobial host specificity is at the sub-species level or strain level [4]. Therefore, specific and rapid analyses of rhizobia at species and sub-species levels can significantly promote biological nitrogen fixation research and applications of rhizobia in agriculture and grassland eco-system management [5,6].

Bacteria are classified into genus and species, by which allows for successful identification of new isolates. Many methods are used to identify bacteria. These include cultural methods (media, morphology, antibiotic and other biochemical tests), serological methods, bacteriophage typing, molecular biology methods (nucleic acid hybridization and PCR-based techniques) and genomic sequencing [7–11]. However, no single method has no drawbacks. The possibility of identifying bacteria using mass spectrometry (MS) was discussed in the early 1970’s [12], and various roles of MS in detection and characterization of microorganisms were proposed later [13]. MS was then introduced to rapidly identify intact microorganisms [14]. With the development of proteomics and bioinformatics, protein databases were demonstrated successfully to support MS identification of microorganisms [15]. 16S rDNA gene sequencing was also compared with MS for species identification of nonfermenting bacteria [16]. Teramoto et al. [17] identified bacteria by using 50 subunit ribosomal proteins. Saffer et al. [18] used matrix-assisted laser desorption/ ionization time-of-flight MS (MALDI TOF MS) and BioTyper™ to have correctly identified 408 and 360 gram-negative bacilli strains at the genus and species levels at a successful rate of 93% and 82%, respectively.

The MS technique for rapid identification and classification of microorganisms has attracted great interests from microbiologists for applications in rhizobial research [19]. MALDI TOF MS showed to be a fast and reliable platform for identification and ecological studies of species from family *Rhizobiaceae* [20]. MALDI TOF MS was also applied for in situ identification of plant-invasive bacteria, e.g., rhizobia in nodules [21]. However, the MALDI TOF MS technique requires a well-established reference spectral database for accurate bacterial identification [22]. The sample preparation and growth period of bacteria such as rhizobia also affect the quality and reproducibility of the protein mass spectra [23].

In the present study, *Escherichia coli* DH5α and well-characterized type strains of four rhizobial species were chosen as reference strains to investigate bacterial cultivation, colony storage conditions and sampling time for quality and consistent MALDI TOF MS spectra and profiles for accurate identification of species of rhizobia. The optimized conditions were used to culture 75 rhizobial strains of 4 genera for which a MALDI TOF mass spectral library was constructed and validated with a blind sample. This is a first step to build a protein profile library for identification and classification of species of rhizobia.

## Materials and Methods

### Chemical reagents and solvents

All chemicals were purchased from Sigma-Aldrich (St. Louis, MO, USA), Fisher Scientific (Pittsburgh, PA), or Alfa Aesar (Ward Hill, MA, USA). All organic solvents and water (LC MS grade) were purchased from Fisher Scientific. The matrix α-cyano-4-hydroxycinnamic acid (HCCA) was bought from Brucker Daltonics (Billerica, MA, USA). All pipette tips and tubes were bought from Eppendorf (Hauppauge, NY, USA).

### Bacterial strains and cultivation

Rhizobial strains were listed in Table 1. The rhizobial strains that were published included *Rhizobium miluonense* [24], *Rhizobium multihospitium* [25], *Mesorhizobium septentrionale, Mesorhizobium temperatum* [26], *Mesorhizobium gobiense and Mesorhizobium tarimense* [27]. All other genera and species were obtained from the Collection Center of Beijing Agricultural University (CCBAU, Beijing China). Yeast-Mannitol-Agar (YMA) or YM broth without agar (YMB) media were used for rhizobial cultivation according to Vincent’s method [28] and Luria-Bertani (LB) media for *E. coli*. *E. coli* DH5α as the standard reference strain was available from our previous work [29].

### Growth curve of reference strains

To study the influence of growth phase on MALDI TOF MS protein profiles, triplicate bacterial samples from early exponential phase, exponential and log phases in liquid medium were analyzed. *Bradyrhizobium yuanmingense* CCBAU 10071^T^, *Mesorhizobium tianshanense* USDA 3306^T^, *Rhizobium leguminosarum* USDA 2370^T^ and *Sinorhizobium meliloti* USDA 1002^T^ were selected to represent the four rhizobial genera and were cultured in 20 mL of liquid YMB medium in a 150 mL flask at 28°C and 150 RPM on a shaker incubator. Aliquots of the cultures of the fast-growing *R. leguminosarum* USDA 2370^T^ and *S. meliloti* USDA 1002^T^ were sampled at 10, 18, 24, 30, 48 and 72 h. Aliquots of the cultures of the slow-growing *M. tianshanense* USDA 3306^T^ was sampled at 18, 30, 48, 72 and 96 h, while *B. yuanmingense* CCBAU 10071^T^ was sampled at 24, 30, 48, 72, 96 and 144 h. *E. coli* DH5α was cultured in 20 mL of liquid LB medium in a 150 mL flask at 37°C and 150 RPM on a shaker incubator, and was sampled at 3, 6, 9, 12, 18, 24 and 30 h. The cell concentrations were determined spectrophotometrically at OD600 nm (Varian Cary 50, Agilent Technologies, Santa Clara, CA, USA). The samples were adjusted to 1 × 10^8^ cfu/mL by centrifugation at 6000 × *g* and dilution. All samples were further washed by TES solution (Tris- EDTA-Sodium chloride) to remove the media and polysaccharides. At each time course, three biological replicates were harvested for protein extraction prior to MALDI TOF MS analyses.

### Storage and duration of reference strains

To study the influence of colony storage temperature and duration on MALDI TOF MS protein profiles, pre-grown colonies on solid medium were stored at either 4 or 21°C for up to 3 weeks and then analyzed. The strains CCBAU 10071 and USDA 3306 were cultured in the YMA medium for 7 d, while the strains USDA 2370 and USDA 1002 for 3 d. *E. coli* DH5α was cultured in the solid LB medium for 1 d. The pre-well-grown *E. coli* colonies were stored at 4 or 21°C for 0, 2, 7 and 14 d. The well-grown rhizobial colonies were stored at 4 or 21°C for 0, 7, 14 and 21 d. During the storage, about 100 mg of bacteria colonies with three biological replicates was sampled and stored for protein extraction prior to MALDI TOF MS analyses.

### Strains used to build an in-house protein mass spectral profile library

The optimized method was further employed to identify and classify 75 rhizobial strains in 40 characterized species in 4 genera (6 *Mesorhziobium* spp., 22 strains; 9 *Rhizobium* spp., 49 strains; 3 *Sinorhizobium/Ensifer* spp., 3 strains; and 1 *Azorhizobium* sp., 1 strain). The potential of developing a mass spectral database for determination of rhizobial species was demonstrated with blind samples.

### Protein extraction

Proteins were extracted according to the published methods [30]. Specifically, approximately 5–10 mg or up to 1×10^8^ cfu of fresh cells was collected in an Eppendorf tube. An aliquot of 300 µl water was added to resuspend the pellet followed by addition of 900 µl ethanol. The tube was vortexed for 1 min and then centrifuged at 15000 × *g* for 2 min. After the supernatant was discarded, the process of addition of 300 µl water and 900 µl ethanol, vortexing and centrifugation was repeated once again. After the supernatant was completely discarded, 50 µl of 70% aqueous formic acid (v/v) and 50 µl of acetonitrile were added and vortexed well. After centrifugation at 15000 × *g* for 2 min, the protein supernatant was transferred to a new tube and then kept at −20°C or immediately used in the next step. Each sample had three biological replicates.

### Matrix preparation

Saturated HCCA solution was prepared in 50% aqueous acetonitrile containing 2.5% tri-fluoroacetic acid (v/v) at room temperature and sonicated for 10 min. The solution was stored at 4°C. Before use, the saturated HCCA solution was sonicated for 5 min and then centrifuged at 5000 RPM.

### MS target plate preparation

An amount of 1 µl sample was directly spotted on a stainless steel target plate (MTP 384 target ground steel, Bruker Daltomics, Germany) and dried under a gentle stream of nitrogen gas, followed by addition of 2 µl of the HCCA solution on the dried sample spot. The plate was dried again under the nitrogen gas prior to MALDI TOF MS analysis.

### Instrument data acquisition

The Bruker UltraFlex TOF/TOF III was calibrated for the analysis of each batch of samples. The standard tuning procedures were first performed by using Protein Calibration Standard I (Bruker Daltonics). MS measurement of *E. coli* DH5α was then served as a positive control to confirm proper sample preparation, instrument tuning and calibration. It is important to generate an *E. coli* mass spectrum that includes the peak at 10299.09 Da. This is a characteristic peak for *E. coli* DH5α, indicating proper sample preparation, instrument tuning, calibration and operation.

The samples were analyzed with a Bruker UltraFlex TOF/TOF III in linear mode, positive polarity, ion source 1 at 25 kV, ion source 2 at 23.6 kV, and lens at 6.5 kV. Six mass spectra were obtained from each sample spot, and each spectrum was accumulated from 100 laser shots. The raw spectra were patch-processed by FlexAnalysis (v3) with a default value using the protein mass fingerprint flex analysis mass spectrometry (PMF.FAMS) method. The summed spectra that were acquired were processed and annotated within the mass range of 300–15000 m/z. The raw spectra were subsequently analyzed by MALDI BioTyper™ v1.1 (Bruker). In total, 18 mass spectra were generated from 3 biological replicates and 6 technical repeats for each sample.

### Data analysis

Bacterial growth variations were analyzed by ANOVA (SAS v.8.0, SAS Institute Inc., Cary, NC, USA). The spectrum peak lists (protein list) were generated by setting at 3000–15000 m/z, with resolution at 1 Dalton (Da), and the mass was adjusted using the spectra compressing method with a compression factor of 10 to reduce and remove high frequent spikes. The baseline was smoothed by the Savitsky-Golay method with a 25 Da frame size for one smoothing polynom and normalized by the maximum norm method by setting the highest peak to the value “1” and calculating all other peaks in respect to the highest peak, which were corrected twice by calculating and constructing by approximating multiple polygons to the spectrum. The peaks were searched by, the Peak Fitting method with Gauss profile to fit a reconstructed spectrum by a peak model. The maximum number of peaks was 100, which the peaks had an intensity at least 5% of the highest peak (i.e., threshold of 0.05).

Composite correlation index (CCI) was used to determine the relationships between two spectra. A CCI-value of 1 represents the highest conformity of the spectra, while CCI-values near 0 indicate a clear diversity of the spectra. A CCI-value greater than 0.9 indicates a good conformance with the reference species [31]. Score or Log (score) values were calculated in the identification process by comparing the unknown sample spectra with peak lists of the main spectra, followed by peak alignment and spectral calibration. It was suggested that a Log (score) of 2 or greater represents the same species [32,33]. However, other authors suggested that a Log (score) of ≥ 1.7 was considered a high confident identification [34]. In the present study, we adopted identification criteria recommended by the MS and BioTyper manufacturer and Jamal et al. [35] and Spanu et al. [36]; specifically, scores of 0.00–1.699 represent no reliable identification, scores of 1.700–1.999 were considered as probable identification at the genus level, scores of 2.000–2.299 as secure genus identification and probable species identification, and scores of 2.300–3.000 as secure identification at species level. In other words, the manufacturer’s recommended cut-off scores were used to determine the genus-level (1.700–1.999) and species-level (≥ 2.000) identification.

To investigate effects of growth status and duration on protein profiles, mass peak lists were converted into binary numbers (1 or 0) based on the presence or absence of the mass peak at a resolution of 1 Da. The number (n) of peptide fragments occurring in each mass segment as peptide richness was recorded and analyzed according to the correspondence analysis procedure (PROC CORRESP, SAS Inc., USA).

For each strain, the peak list of all replicates was re-constructed into a main spectrum to represent the strain mass profiles. Dendrograms were calculated by a distance method with the unweighted pair group method with arithmetic mean (UPGMA) via PAUP 4.0b10 [37] to compare the 16S rDNA phylogenetic tree.

Protein mass spectral libraries of the rhizobia at species and genus levels were constructed according to the MALDI BioTyper™ manual. Briefly, each species-level library spectrum was reconstructed from the spectra of all strains within the species, e.g., the protein mass spectrum of each strain was reconstructed from 3 biological replicates and 6 technical replicates (3 × 6). Each genus-level library spectrum was reconstructed from all protein mass spectra of all species within the genus. The species of a blind sample was identified by comparing its protein mass spectrum with those in the genus and species libraries.

## Results

### Effects of growth phase, colony storage conditions and duration on *E. coli* DH5α protein mass spectral profiles

**Figure 1:**
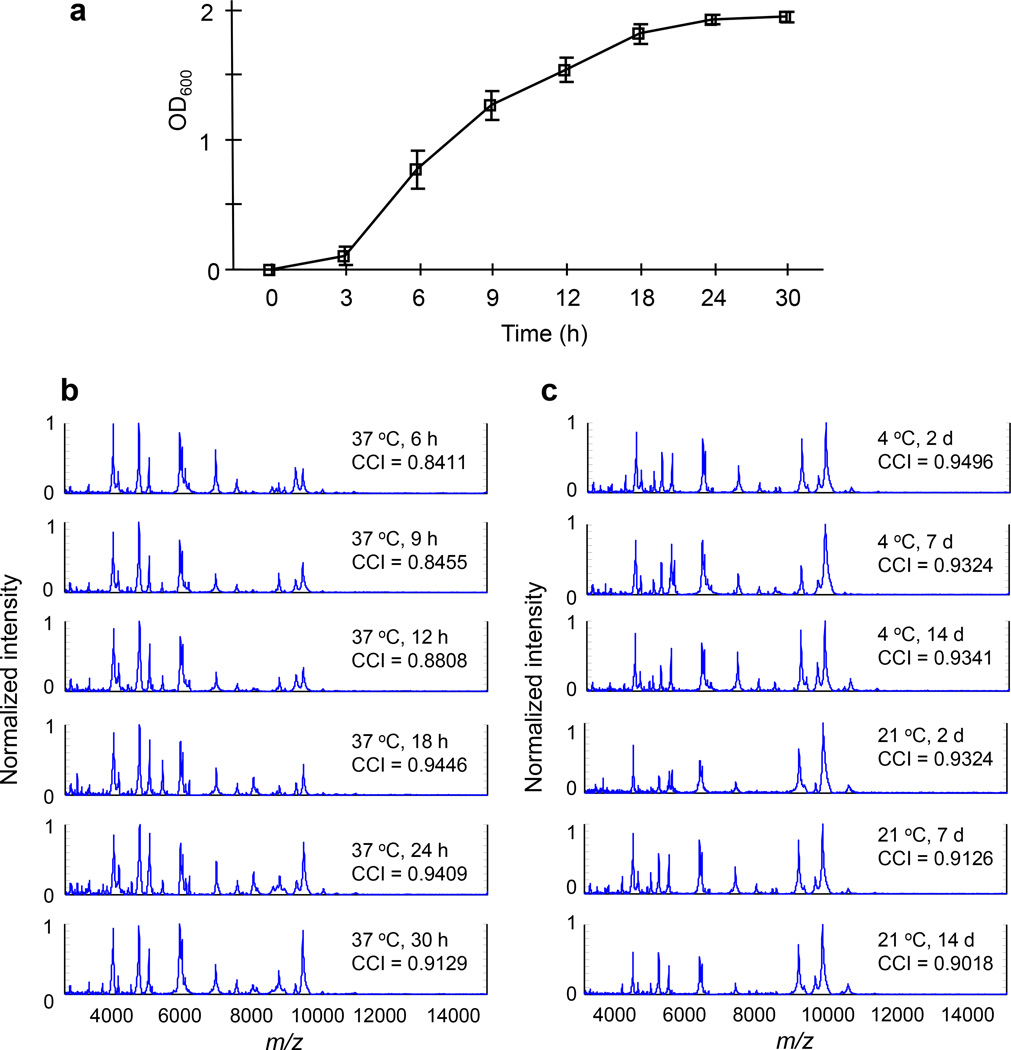
*E. coli* growth curve and mass spectral profiles Growth curve of *E. coli* DH5α at 37°C (a), mass spectra of *E. coli* whole cell proteins at different growth stages (b) and storage at 4°C or 21°C (c), CCI indicates the composite correlation index.

Protein mass spectral profiles of *E. coli* during each growth phase were measured to assess the impact on bacterial identification (Figure 1). *E. coli* DH5α cells cultured for 3, 6 and 9 h, which were in the early exponential phase, had low CCI values of 0.8411, 0.8455 and 0.8808, respectively. The cells in the exponential and post-exponential phases had CCI values of 0.9446 (18 h) and 0.9409 (24 h), respectively, while the CCI value remained at 0.9129 in the stationary phase (30 h). The colony storage temperature (4 and 21°C) and duration (2, 7 and 14 days) did not dramatically affect *E. coli* identification. The CCI values were 0.9496, 0.9324 and 0.9341 after the colonies were stored at 4°C for 2, 7 and 14 days, respectively. The CCI values of the cells stored at 21°C for 2, 7 and 14 days were 0.9324, 0.9126 and 0.9018, respectively. Storage temperature had no significant impact on the protein profiles even up to 2 weeks of storage.

### Effects of growth phase, colony storage temperature and duration on protein profiles of the four well characterized rhizobia reference strains

**Figure 2:**
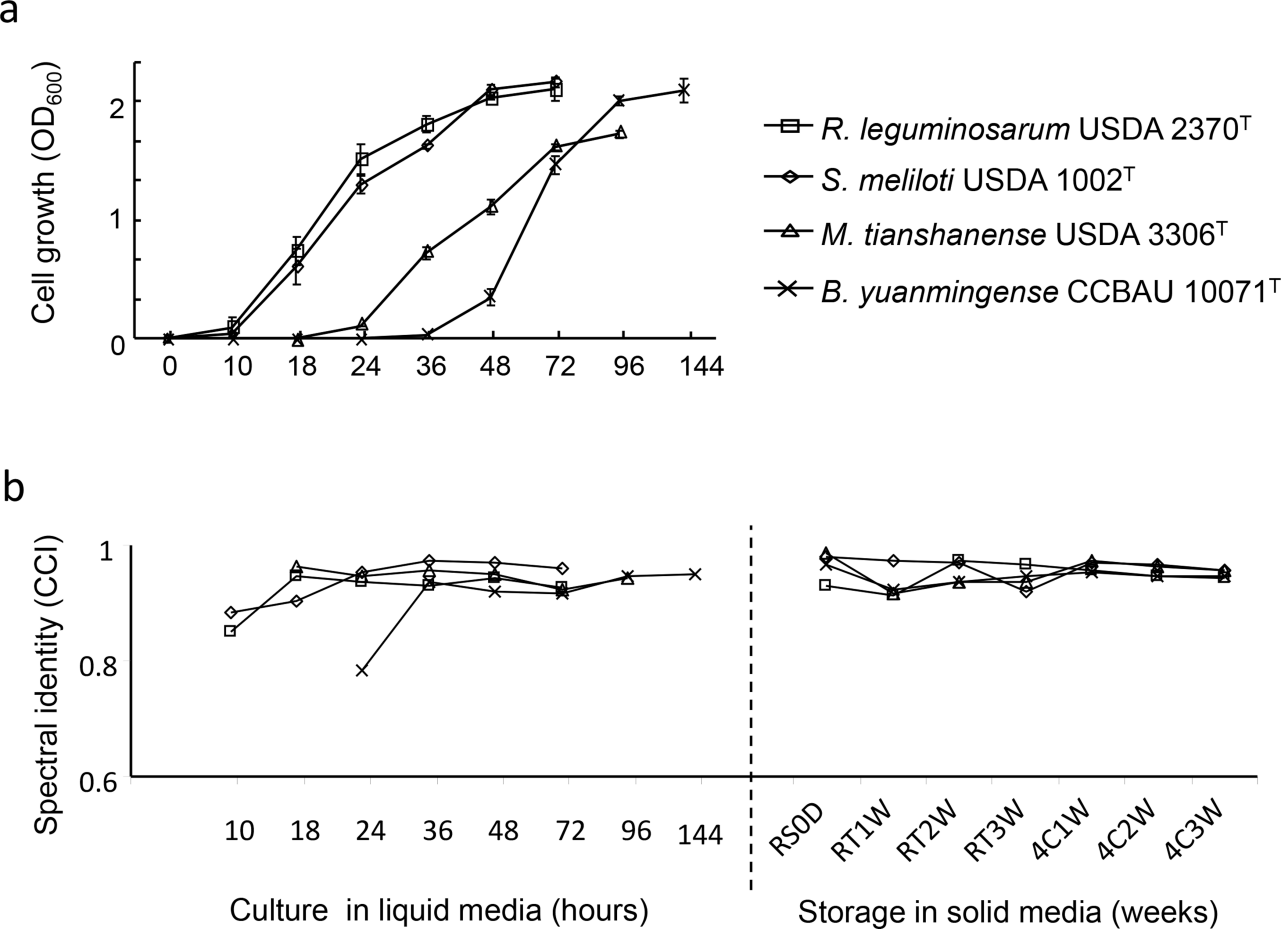
Rhizobia growth curve and mass spectral identity. (A) Cell growth curves and (B) spectral identities (CCI value) of rhizobial strains *S. meliloti* USDA 1002^T^, *R. leguminosarum* USDA 2370^T^, *M. tianshanense* USDA 3306^T^, and *B. yuanmingense* CCBAU 10071^T^ at different growth stages and storage periods. RS0D: pre-well grown media marked as 0 day, RT indicates room temperature (21 °C) storage, 4C indicates 4 °C storage. 1W – 3W means 1 to 3 weeks.

As was the case for *E. coli*, all four rhizobial strains in the exponential and post-exponential phase had highly consistent identity (Figure 2). The cell number of the fast growing *R. leguminosarum* USDA 2370^T^ and *S. meliloti* USDA 1002^T^ at first 10 h of inoculation was not significantly changed or was slightly increased, giving less consistent spectra (CCI 0.883 and 0.851, respectively). Cells from exponential and post-exponential phases (18–36 h) had highly consistent spectra, which the CCI values for USDA 2370^T^ and USDA 1002^T^ were in a range of 0.903–0.972 and 0.938–0.942, respectively. When the USDA 1002^T^ and USDA 2370^T^ cells were in the stationary phase (48–72 h), the spectra maintained high consistency (CCI 0.969–0.961 and 0.942- 0.928, respectively). The slow growing *B. yuanmingense* CCBAU 10071^T^ also produced less consistent spectra (CCI, 0.782) at the early exponential phase (36 h), but had high CCI values (0.916–0.951) from the exponential phase to the stationary phase. The slow-moderate growing *M. tianshangenese* USDA 3306^T^, from the early exponential phase (18–24 h) to the stationary phase (72–96 h), differed from the other three strains in that it always maintained relatively high spectral consistency (average of CCI, 0.948).

### Mass spectral profiles of the four well characterized rhizobia reference strains at different growth stages and storage durations

**Figure 3:**
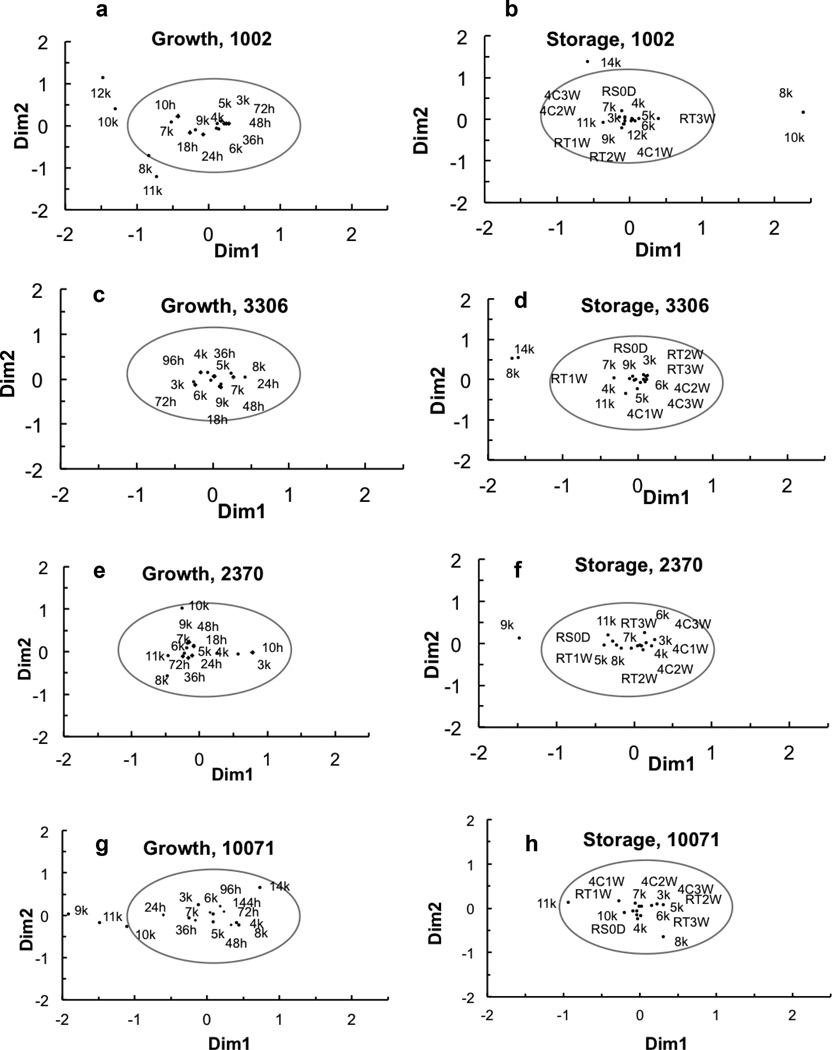
Analyses of mass spectral profiles of proteins of the four well characterized rhizobia reference strains at different growth stages and storage durations. *S. meliloti* USDA 1002^T^ growth (a) and storage (b), *M. tianshanense* USDA 3306^T^ growth (c) and storage (d), *R. leguminosarum* USDA 2370^T^ growth (e) and storage (f), and *B. yuanmingense* CCBAU 10071^T^ growth (g) and storage (h). 3K–14K stands for all mass fragments by 1000 m/z. 10 h, 18 h, 24 h … 144 h stand for bacterial growth times in hours. RS0D stands for the well-grown fresh bacteria (0 day). 1W, 2W and 3W stand for the storage duration of 1, 2 and 3 weeks, respectively. 4C stands for the storage temperature 4°C. RT stands for room temperature (21°C). Dots represents protein mass segment, diamonds represents different growth time (10–144 h), squares represent different storage method and duration. Dimension 1 (Dim1) and dimension 2 (Dim2) explained the variation of proteins distribution among the treatments.

Mass spectral profiles of abundant proteins (mostly ribosomal proteins) in bacterial cells at each time point were compared to evaluate the resolution, accuracy and reliability of bacterial identification with different storage and growth conditions (Figure 3). To minimize interferences caused by growth stage and storage, correspondence analysis was performed by dividing the 1000 m/z (1K) into different segments. Protein profiles of *S. meliloti* USDA 1002^T^ showed similar results of CCI evaluation of spectral identity (Figure 3a and 3b). Proteins from the cells that grew in the liquid culture in the mid-/post-exponential phase (24 h and 36 h) and stationary phase (48 h and 72 h) tightly clustered together, while they in the early exponential phase (10 h and 18 h) remained within the cluster with slight variations. The distribution of peptide fragments in the segments 3K to 7K varied much less relative to those in the segments 8K, 10K to 12K. Peptide fragments in the 13K and 14K segments were not detected over the entire growth phase. Protein profiles of USDA 1002^T^ did not show any significant changes with up to 3 weeks of storage at either 4 or 21°C (Figure 3b). Protein fragments distributed in the 3K–7K and 9K were constitutive, whereas protein fragments in the 8K, 10K, 11K, 12K and 14K varied during growth and storage.

The growth stages of USDA 3306^T^ did not affect protein fragment patterns (Figure 3c). The fragments were distributed in 3K–9K segments. The protein profiles were quite stable for the cells stored up to 3 weeks at either 4 or 21°C. In addition to the proteins occurring in the segments 3K–9K, proteins were detected in segments 11K and 14K when grown on solid media. When USDA 3306^T^ colonies were stored at 4 and 21°C for up to 3 weeks, all proteins except those in the segments 8K and 14K clustered closely (Figure 3d).

Protein fragment distributions of USDA 2370^T^ varied little for the cells analyzed between mid-exponential phase and stationary phase (18–72 h) (Figure 3e). Protein fragments in the 5K–7K, 9K and 11K segments were constitutive, while protein fragments were not observed in the segments of 12K–14K. Protein profiles of USDA 2370^T^ cells varied little during storage up to 3 weeks (Figure 3f). Protein fragments within the segments of 3K–8K and 11K remained constant during storage at either 4 or 21°C, but fragments in segments 9K and 14K varied. Protein fragments in segments 11K–13K were not detected.

The mass spectral profiles of proteins of *B. yuanmingense* CCBAU 10071^T^ at different growth stages were less clustered than those of USDA 1002^T^, USDA 3306^T^ and USDA 2370^T^ (Figure 3g), which indicates some effects of culture duration on the whole cell protein profiles. Protein fragments 3K–8K were clustered, while those in the 9K–11K and 14K were out of the cluster, indicating large variations of the composition of proteins at the different growth stages. Those less consistent protein fragments might play minor roles in bacterial identification. The storage of colonies at 4 or 21°C did not affect the protein patterns (Figure 3h).

The multiple correspondence analyses showed that the colony storage time and temperature, for up to 3 weeks, have little effects on the protein fragment distribution. Cultivation with either liquid or solid media did not significantly affect identification. Dividing protein fragments into different segments allows comparison of highly conserved proteins, being relevant to bacterial identification, with non-conserved proteins which changed during bacterial growth or storage. The results also confirmed that the most suitable sampling time for identification was the post-exponential or stationary phase. Samples tested were successfully identified after 3 weeks of storage at 21°C or 4°C, which validated this method.

### Dendrograms of protein mass spectral profiles and 16S rDNA phylogenetic tree

**Figure 4:**
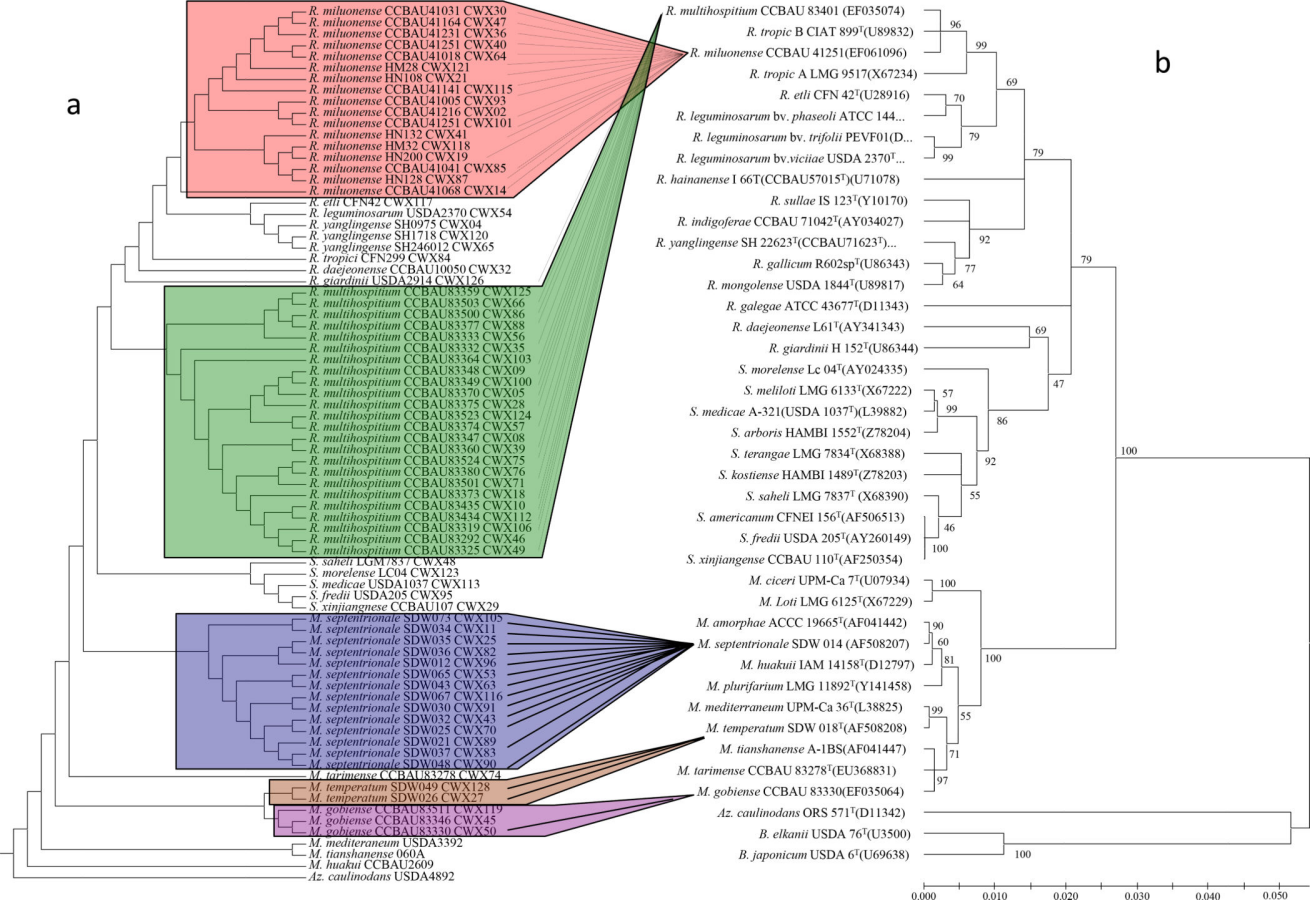
Comparison of the dendrogram of whole cell protein profiles and 16S rDNA-based phylogenetic trees of rhizobia. a) The dendrogram was generated via PAUP 4.0b package. Briefly, totally 15–18 replicates of protein peak lists (3 biological replicates and 6 technical replicates from each sample) were combined together if the same protein peaks repeated greater than 95% of chances in all 15–18 replicates that were counted. Each sample was, thus, assigned to “mainspectra peak lists”. Subsequently, we coded all the sample’s peak lists into a binary classifier system (present 1, absent 0). The generated main data matrix was used to perform phylogenetic analysis via PAUP 4.0b. The distance matrix tree was calculated with UPGMA method, with 1000 bootstrap value. b), the phylogenetic tree was generated using full length of 16S rDNA sequences, the tree was also calculated with UPGMA method by using MEGA 5.0 package. An accession number (GenBank No.) was listed. The UPGMA tree was tested with the bootstrap method (bootstrap replicates 1000). The percentage of replicates in which the associated taxa clustered together in the bootstrap test is shown next to the branches. The value less than 50 indicated the unstable clade and the branches might collapse. The substitution model used nucleotide type and maximum composition likelihood method. The branches unit is the number of base substitution per site.

In comparison with 16S rDNA, protein dendrograms showed a high resolution at levels of genera, species and strains (Figure 4a). All strains within a species were clustered closely. The protein dendrogram shared the similar topology with the 16S rDNA phylogenetic tree (Figure 4b).

### Identification of a blind sample via a two-step strategy

**Figure 5:**
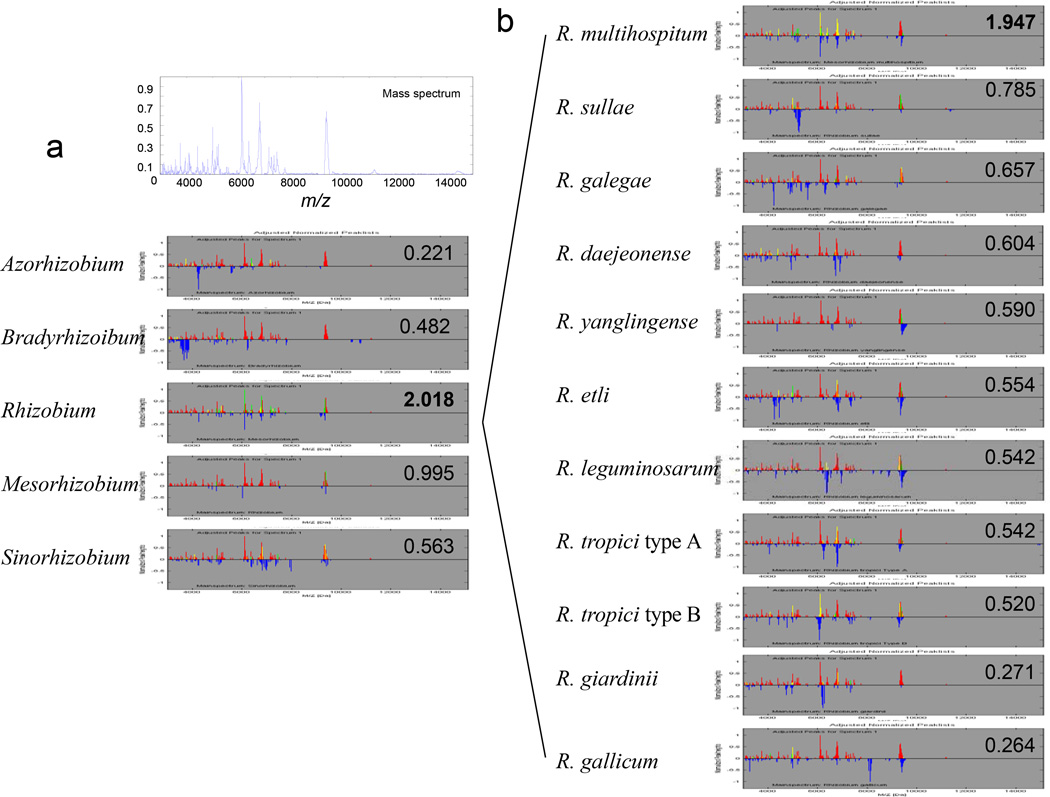
Two-steps for blind sample identification (a) Identification of a blind strain via protein mass spectral profiles at genus level. (b) Identification of rhizobia via protein mass spectral profiles at species level.

A tiered strategy, i.e., two steps in the present study, was followed to identify the species of a randomly-selected blind sample to evaluate the main spectrum library. The first step was identification at the genus level. All strains in a genus were used to build the genus libraries of *Azorhizobium, Bradyrhizobium, Mesorhizobium, Rhizobium* and *Sinorhzobium*. The blind spectrum matched well with that of a *Rhizobium* strain in the library, having the highest Log (score) 2.018 (Figure 5a), but not with those of the strains in the genera *Mesorhizobium* (0.995), *Sinorhzobium* (0.563), *Bradyrhizobium* (0.482), and *Azorhizobium* (0.221). The second step was identification at the species level. The blind sample spectrum matched with those in the species library. The highest Log (score) 1.947 matched with *R. mutlihosptium* (Figure 5b), while the blind sample spectrum matched with other *Rhizobium* species in a range of Log (score) from 0.264 to 0.785. The identification result agreed with the previous study [24].

## Discussion

Our results showed high stability and reproducibility of the whole cell protein profiles in liquid and solid media and a suitable sampling time between the exponential and stationary phases. *E. coli* colonies could be stored up to 2 weeks. This is important in situations where immediate analysis is not feasible. *Erwinia* bacteria samples from agar media (solid) or liquid media gave very good mass spectra in terms of sensitivity and resolution [38]. The bacteria samples could be stored at room temperature for several days or 4–8°C for several weeks [38]. A proper protein extraction method also enhanced the quality of the mass spectra [39].

The mass spectra of both *E. coli* and rhizobia with different growth-rates were not affected by media type (solid or liquid). Different growth stages except for the log and early exponential phases had negligible effects on consistency of the mass spectra. Two different storage temperatures (4°C and 21°C) using solid media were tested to determine the best sampling conditions. The results showed that all four strains maintained high spectral consistency for up to 3 weeks at both 4°C and 21°C, and there was no indication that 4°C was better than 21°C. Such results suggest that this technology has a high tolerance of variations caused by different growth and storage conditions. MS, as an easy, rapid, high throughput, and efficient identification technique for clinical diagnostic laboratories, has the potential to complement conventional identification of bacteria, and as such storage would be less of an issue [40].

Fine-tuned mass spectra of ribosomal proteins would enhance the identification confidence. In addition to use of a high-quality reference database, distinct mass spectral pattern and annotation of the characteristic ribosomal proteins by molecular weight enhanced the resolution of clostridial identification [41]. Comparing the peaks with the reference ribosomal subunit protein peaks to generate a dendrogram would be sufficient for the taxonomic analysis without using DNA sequence information [42]. Intact *E. coli* ribosomes were studied with nanoflow electrospray ionization MS to investigate the dynamics and state of specific proteins [43]. The peaks generated were essential for accurate classification and identification of bacteria [30].

Bacterial classification is often a time and resource consuming process [44]. Genome sequencing has a potential application in the future of bacterial taxonomy. However, there is no consensus as to bacterial classification, partly because alignment and analysis of large numbers of conserved genes have given inconsistent results [45], and also it may be further complicated by the presence of horizontal gene transfer [46]. In the past decades, efforts have been made to characterize a species using MS and computer technology complement to the phenotypic and genotypic description of bacteria. A significant advantage of such an approach is the technology’s high resolution.

Through MS studies, we can observe detailed information as to the protein distribution with different growth stages and storage conditions. In the present work, we applied 1KDa “segments” for the protein profiles, which showed that different biological states affected the protein profiles. Thus, we proposed a two-step approach to identify an unknown species. An advantage of this method is that “genus” markers would be stable among species in the same genus. The loci of the test strains can be readily narrowed to finally identify the target strain at a species level. Successful and accurate identification of bacteria was achieved using available databases. In most cases, lack of identification or erroneous identification was due to improper database entries [47]. Accurate MALDI TOF MS identification was significantly correlated with having 10 reference spectra in the database [47]. A dedicated reference set and spectra of high quality are required to unambiguously identify *Burkholderia* species [48]. A principle of the technology means that highly abundant proteins (mostly ribosomal proteins) will contribute more to the identity of a strain, while some protein composites may be altered as they are more “sensitive” to growth, storage, or stress conditions. Careful creation of reference spectra databases [48] in future applications would offer high accuracy to offset the 16S rDNA sequencing and DNA-DNA hybridization in bacterial classification and identification.

The results of rhizobial identification and classification using MALDI TOF MS can be summarized in seven points. (1) The state of the media (solid versus liquid) was not a significant factor in determining rhizobial protein profiles. (2) The best sampling time was the exponential phase and post-exponential phase. (3) Satisfactory identification was achieved with samples stored at 4°C or 21°C for up to 3 weeks. (4) Not all proteins detected by MS were constitutively-expressed, and those proteins’ presence and absence varied among the species. (5) Multiple MS data acquisitions from the same sample, as well as those from multiple strains in the same species, will refine the protein profiles and reference library. (6) Identification based on protein mass spectral profiles was primarily consistent with that obtained by 16S rDNA. (7) The two-step identification approach allows the sample to be first identified at a genus level, and then at a species- or sub-species level. The process from protein extraction to species identification required approximately 60 min.

## Figures and Tables

**Table 1:** Bacterial strains used in the present study.

Species	Strains
***Mesorhizobium***	
*M. amorphae*	B276^T^ (ACCC 19665T)^1^, ACCC19663, sh190012, ACCC19660
*M. septentrionale*	SDW014^T^, SDW012, SDW033, SDW034, SDW035, SDW036, SDW073, SDW048, DW020, SDW028, SDW030, SDW065, SDW067, SDW068, SDW043, SDW047, SDW044, SDW032, SDW037, SDW021, SDW025, SDW060, SDW064
*M. temperatum*	SDW015, SDW016, SDW018^T^, SDW039, SDW050, SDW055, SDW026, SDW038, SDW049, NM026, NM300
*M. gobiense*	CCBAU 83330^T^, CCBAU 83346, CCBAU 83511
*M. tarimense*	CCBAU 83306^T^, CCBAU 83321, CCBAU 83278
*M. chaoconense*	LMG 19008^T^
*M. ciceri*	USDA 3378^T^
*M. multihospitium*	CCBAU 83401^T^, CCBAU 83345, CCBAU 83319, CCBAU 83325, CCBAU 83347, CCBAU 83348, CCBAU 83349, CCBAU 83277, CCBAU 83373, CCBAU 83374, CCBAU 83375, CCBAU 83376, CCBAU 83377, CCBAU 83380, CCBAU 83370, CCBAU 83332, CCBAU 83333, CCBAU 83283, CCBAU 83292, CCBAU 83359, CCBAU 83360, CCBAU 83364, CCBAU 83434, CCBAU 83435, CCBAU 83500, CCBAU 83501, CCBAU 83503, CCBAU 83504, CCBAU 83523, CCBAU 83524, CCBAU 83525
*M. huakui*	CCBAU 2609^T^
*M. loti*	NZP 2213^T^
*M. mediteraneum*	USDA 3392^T^
*M. plurifarium*	LMG 11892^T^
*M. tianshanense*	A-1BS^T^ (CCBAU 3306)^2^, CCBAU 6, CCBAU 017A, CCBAU 032B, CCBAU 060A, CCBAU 005B, CCBAU 009B, CCBAU 91X01
***Rhizobium***	
*R. etli*	CFN 42^T^
*R. galegae*	USDA 4128^T^ (HAMBI540)
*R. gallicum*	USDA 2918^T^
*R. giardinii*	USDA 2914^T^ (H152)
*R. leguminosarum*	USDA 2370^T^
*R. mongolense*	USDA 1844^T^
*R. sullae*	USDA 4950^T^ (IS123)
*R. tropici typeA*	CFN 299^T^
*R. tropici typeB*	CIAT 899^T^ (LMG9503)
*R. daejeonense*	CCBAU 10050^T^ (IAM 15042)
*R. yanglingense*	CCBAU 71462 (sh246012), CBAU 71012 (sh17113), CCBAU 71078(sh1718), CCBAU 71623T (sh22623), CCBAU 71060 (sh1456), CCBAU 71152 (sh28931), CCBAU 71036 (sh0975)
*R. indigoferae*	CCBAU 71042^T^, CCBAU 71260 (SH712), CCBAU 71064
*R. miluonense*	CCBAU 41108, CCBAU 41114, CCBAU 41128, CCBAU 41068, CCBAU 41018, CCBAU 41005, CCBAU 41132, CCBAU 41200, CCBAU 41031, CCBAU 41126, CCBAU 41164, CCBAU 41251^T^, CCBAU 41041, CCBAU 41231, CCBAU 41216, CCBAU 41141
***Sinorhizobium***	
*S. arboris*	HAMBI 1552^T^
*S. fredii*	USDA 205^T^, CCBAU 103 (RX11)
*S. kostiense*	HAMBI 1489^T^
*S. kummerowiae*	CCBAU 71714^T^
*S. medicae*	USDA 1037^T^ (A321)
*S. meliloti*	USDA 1002^T^
*S. morelense*	Lc04^T^ (LMG 21331)
*S. saheli*	USDA 4102^T^
*S. terangae*	USDA 4101^T^
*S. xinjiangnese*	CCBAU 110^T^, CCBAU 109 (RX41), CCBAU 108, CCBAU 107 (RX31), CCBAU 105 (RX22)
***Bradyrhizobium***	
*B. elkanii*	USDA 76^T^
*B. japonicum*	USDA 6^T^
*B. liaoningense*	USDA 3622^T^
*B. yuanmingense*	CCBAU 10071^T^
***Azorhizobium***	
*Az. caulinodans*	USDA 4892^T^
***Escherichia***	
*E. coli*	DH5α

1 Strain numbers in parenthesis are alternative strain numbers (the same strains but in different collection centers).

2 Underlined strains were selected for growth and storage studies.

The superscript letter ^T^ indicates the type strains of the species.
